# Structural Timber Connections with Dowel-Type Fasteners and Nut-Washer Fixings: Mechanical Characterization and Contribution to the Rope Effect

**DOI:** 10.3390/ma15010242

**Published:** 2021-12-29

**Authors:** Manuel Domínguez, Jose G. Fueyo, Alberto Villarino, Natividad Anton

**Affiliations:** 1 Department of Mechanical Engineering, Continuum Mechanics Area, High Polytechnic School of Zamora, University of Salamanca, Avda. Requejo 33, 49022 Zamora, Spain; mdominguez1@usal.es (M.D.); fueyo@usal.es (J.G.F.); 2 Department of Construction and Agronomy, Construction Engineering Area, High Polytechnic School of Ávila, University of Salamanca, Hornos Caleros 50, 05003 Avila, Spain; 3 Department of Construction and Agronomy, Materials Science and Metallurgical Engineering Area, High Polytechnic School of Zamora, University of Salamanca, Avda. Requejo 33, 49022 Zamora, Spain; nanton@usal.es

**Keywords:** structural timber connections, dowel-type fasteners, nut-washer fixings, rope effect, timber mechanical behavior

## Abstract

Dowel-type fasteners are one of the most used type of connections in timber joints. Its design follows the equations included in the Eurocode 5. The problem with these equations is that they do not adequately contemplate the resistive capacity increase of these joints, when using configurations which provoke the so-called rope effect. This effect appears when using threaded surface dowels instead of flat surface dowels, expansion kits or nut-washer fixings at the end of the dowel. The standards consider this increase through a constant value, which is a poor approximation, because it is clearly variable, depending on the joint displacement and because is much bigger, especially when using nut-washer fixings. It is also very important because of the rope effect trigger interesting mechanisms that avoids fragile failures without warning of the joints. For these reasons, it is essential to know how these configurations work, how they help the joint to resist the external loads and how much is the increase resistance capacity in relationship with the joint displacement. The methods used to address these issues consisted of a campaign of experimental tests using actual size specimens with flat surface dowels, threaded surface dowels and dowels with washer-nut fixings at their ends. The resistance capacity results obtained in all the cases has been compared with the values that will come using the equations in the standards. After the tests the specimens were cut to analyze the timber crushings, their widths, the positions and level of plasticizations suffer in the steel dowels and in the washer-nut fixings and the angle formed in the dowel plastic hinges. With all this information the failure mode suffered by the joints has been identified and compared with the ones that the standards predict. The results for the size materials and types of joints studied shows that the crush width average values go from 20 mm with flat surface dowels, to 24 mm in threaded to 32 mm in threaded with washer-nut fixings. The rope effect force/displacement goes from 100 N/m in threaded surface dowels to 500 N/m in threaded with washer-nut fixings. Finally, the load capacities are on average 290% higher those indicated in the standard. The main conclusion is that the rope effect force should be considered in the standards in more detail as a function of multiple variables, especially the displacement of the joint.

## 1. Introduction

Timber structures are formed by pieces joined together in joints, which are of vital importance because they are responsible for transmitting the forces between the members of the structure, so that the whole remains stable [1,2,3]. It is considered that 20% to 25% of the economic cost of the structure is linked to the design and construction of the connections between its different elements, and need a great dedication in time [4]. In addition, it is estimated that in a complete structural calculation the design of the connections can represent up to 70% of the total calculation time for a structure [5]. Another disadvantage added to the use of timber has been the existence of less developed standard than in other materials more used for the construction field, such as steel and concrete [6]. This has been resolved with the entry of normative documents of legal support such as Eurocode 5 [7,8].

One of the typical connections for structural elements of timber are the dowel type joints that generically cover nails, staples, bolts, slingshots and dowel and can be found in a wide range of sizes shapes and materials [4]. The connections with flat surface dowels and without reinforcement elements, such as the fixings with nut-washer at their ends, constitute a type of joint very used in the construction of timber structures with studies in that field. Thus, the timber-to-timber connections with different types of screws and nails were experimentally investigated with the aim to quantify the contribution of their axial resistance to their slip and their lateral load bearing capacity, which is the rope effect in dowel-type timber connections [9,10].

On the other hand, the connections with nut-washers are less common in construc-tion and their investigations are based on the combination of the geometric distribution of the nuts-washers where the aim is also to shorten and simplify the assembly time. In order to improve the load-bearing capacity and rigidity of this type of frame connection [11,12] and even with investigations based on numerical models using FEM simulations [13,14], although they do not deem the contribution that this type of fixings makes to the rope effect. However, the studies with nut-washer fixings to investigate their contribution to the rope effect are more limited and are based on scale joint models, determining the contribution that this effect has in the different failure modes [15].

With all this we must start from the basis that its design is based on the so-called performance theory, which was developed in Europe in the 1940s by Johansen [16], and provides equations for the load limit of connections with different mixed configurations, especially for timber-timber and timber-steel connections [17]. In North America it was also adopted and was called the “European Yield Model” [18].

**Figure A1:**
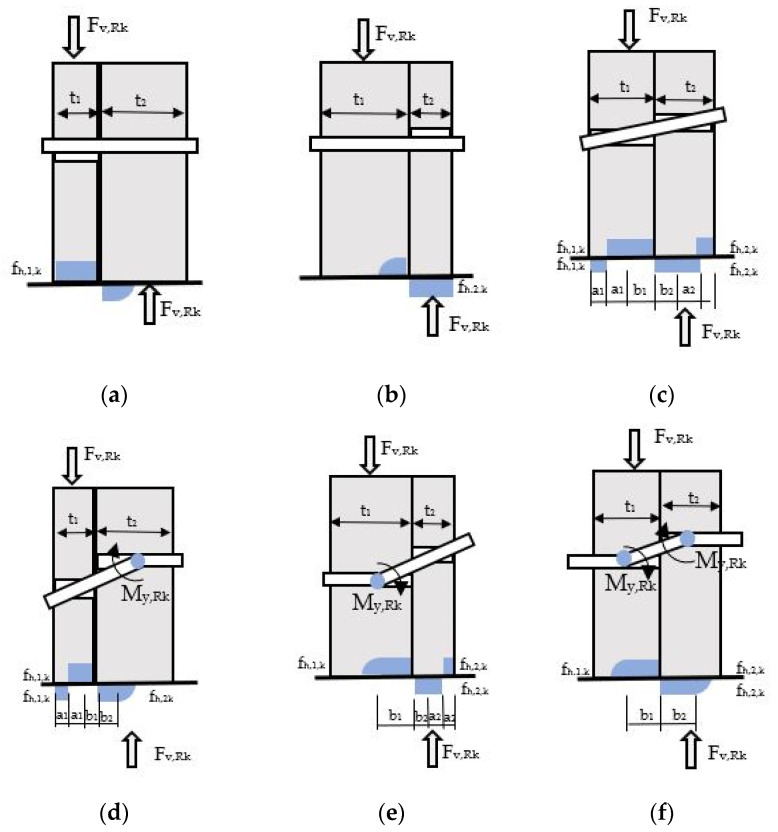
Failure modes in single-shear: (**a**) crushing mode in piece of thickness t_1_; (**b**) crushing mode in piece of thickness t_2_; (**c**) crushing in pieces of thickness t_1_ and t_2_; (**d**) plastic hinge in zone of thickness t_2_; (**e**) plastic hinge in zone of thickness t_2_; (**f**) double plastic hinge in dowel.

The design of connections using these equations was based on studies that analyzed their state of plasticization, considering real size connections and taking into account the main characteristics of the timber and of the elements of the connections, especially the orthotropic behavior of the timber and the failure modes of plastic type, such as the hinges on the dowel or the crushing of certain areas of the timber. Johansen studied the connections considering different hypotheses of tensional distributions according to the type of deformation of the dowel as you can see in Figure A1. Considering the different failure modes, the connection must be designed looking for a ductile failure mode whenever possible [19]. This causes the appearance of failure modes with hinge on the dowel to optimize the work of the joint. In structures, a ductile failure is interesting because it warns of it. The failure modes, which occur with crushing in the timber, force the resizing of the timber section as a result of the fragile failure in the connection [20], and to treat the differences between the gross and net section of the timber to avoid other failures such cracks.These fragile failure modes are related to rigid dowel and affect larger and more expensive parts. For this reason it seems advisable to focus the study on the behavior of the failure mode with double plastic hinge in the dowel.

**Figure A2:**
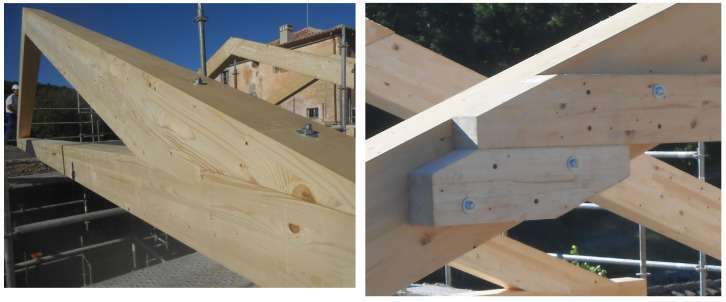
Examples of actual structures using dowel type joints with washer-nut endings.

Using fixings at the ends with nut-washer modifies the load-displacement relationship of the connection by redistributing the crushing stress of the timber and the plastic deformation of the dowel. In addition, the rope effect is contemplated as a direct summation of the load capacity of the connection with a value of 25% of the axial load by rope effect, F_ax,Rk_/4 using Equations (1) and (2) [7]. In the Appendix A examples of applications of these type of fixings in actual structures are shown in Figure A2.

(1) $$F_{v,\mathrm{Rk}3}=1.05\times \frac{f_{h1,k}\times t_{1}\times d}{2+\beta }\times \left[\sqrt{2\times \beta \times \left(1+\beta \right)+\frac{4\times \beta \times \left(2+\beta \right)\times M_{y,\mathrm{Rk}}}{f_{h1,d}\times d\times t_{1}{}^{2}}}-\beta \right]+\frac{F_{\mathrm{ax},\mathrm{Rk}}}{4}$$

(2) $$F_{v,\mathrm{Rk}4}=1.15\times \sqrt{\frac{2\times \beta }{1+\beta }\times }\sqrt{2\times M_{y,\mathrm{Rk}}\times f_{h,1,k}\times d}+\frac{F_{\mathrm{ax},\mathrm{Rk}}}{4}$$

The rope effect increases the resistance by transmission of stresses in the axial direction to the dowel, in a screwed joint because the loads supported by the connection can be distributed towards the screwed ends. For example, when large displacement occurs, the ends of the dowel slide inward, while the nuts and washers resist this movement and are therefore subjected to the opposing action. The rope effect depends on the type and dimensions of the dowel, the density of the timber and the depth of penetration of the dowel into the wood.

From all this, it is observed that the vectors F_v,Rk_ (load capacity at the connection) and F_ax,Rk_ (characteristic value of the load pull out capacity) are vectors that do not necessarily have to have the same direction, in fact, the F_ax,Rk_ modifies its direction depending on the angle of the plastic hinge that could be formed in the dowel as the displacement of the connection increases. Consequently, the contribution of F_ax,Rk_ must be variable depending on the displacement of the connection and not with a constant value corresponding to F_ax,Rk_/4. In addition, the rope effect contribution in the design equations does not appear to provide relevant load capacities since the use of dowel with nut-washer means that a component of a maximum of 25% must be added to the load capacity of the connection. On the other hand, the value of F_ax,Rk_ is calculated taking into account the pull out force using Equation (3) on nails whit treaded surfaces and using Equation (4) for nails with flat surfaces [7].

(3) $$F_{\mathrm{ax},\mathrm{Rk}}=\{\begin{array}{c} f_{\mathrm{ax},k}\times d\times t_{\mathrm{pen}}\\ f_{\mathrm{head},k}\times d^{2}{}_{h} \end{array}$$

(4) $$F_{\mathrm{ax},\mathrm{Rk}}=\{\begin{array}{c} f_{\mathrm{ax},k}\times d\times t_{\mathrm{pen}}\\ f_{\mathrm{ax},k}\times d\times t_{h}+f_{\mathrm{head},k}\times d^{2}{}_{h} \end{array}$$

Factors such as the threading of the dowel, the surface of the washer in contact with the timber and the possible pre-tensioning that increase the rope effect before the connection is loaded are not contemplated. This research analyzes the stress distributed towards the washer-timber contact zone at the ends of the dowel integrating the rope effect as a vector superposition to the resistant capacity of the connection as a whole [21].

## 2. Materials and Methods

### 2.1. Geometry of the Configuration

**Figure 1:**
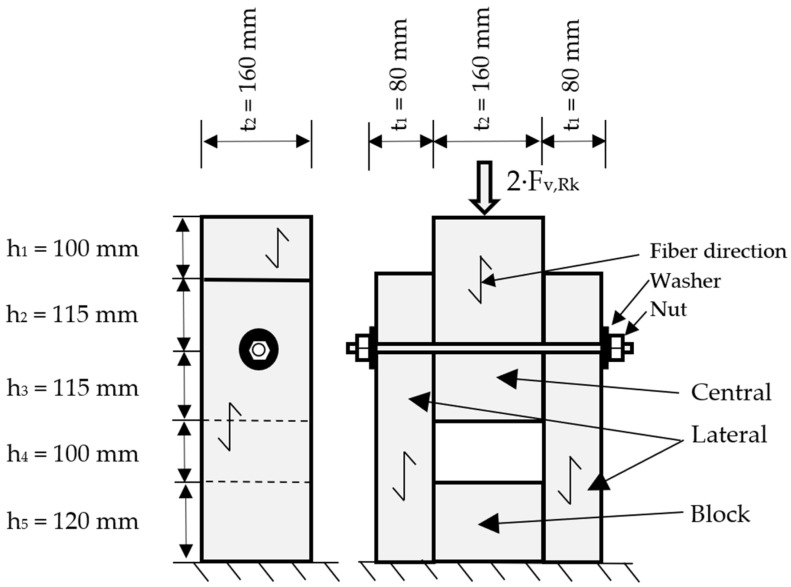
Dimensions and geometry of the type of joint selected for the study.

The case studied involves a dowel-type joint with only a steel element working in double shear. Its configuration, dimensions and boundary conditions are shown in Figure 1. All these variables have been taken from a joint located on the real structure mentioned above.

The tests on dowel-type timber joints pose a complex problem due to the orthotropia and heterogeneity of the timber, as well as the influence of other environmental variables (humidity, time of application of the load, combination of different materials, pathologies, etc.). Generally speaking, the regulations have attempted to make an independent analysis of the failure variables, including crushing of the timber and bending of the dowel. It is usual that the standards of some countries have a clear reference on older standards with recognized experience in this field. In this context, we can cite as examples the ANSI standards of the USA, or the COPANT standards in South America, as a unifying model in this matter [22].

In Europe, the traditional standards belonged to the so-called “basic stress approach” (specimens of small size and, in general, free of defects), based on working with homogeneous materials such as steel and concrete. However, a treatment closer to the reality of structural timber is necessary. For this reason, specimens derived from dimensions typical of real structures have been chosen. Regarding the configuration, among all the possible types of joints in wooden structures with a dowel, the case of joint between pieces of timber in double shear has been chosen, since it is the joint design that transmits the loads in a symmetric way [23]. Figure 1 shows a schematic of the type of joint designed.

### 2.2. Equipment for Caractherization Description

In order to evaluate the behavior and quality of the joint, the materials were characterized. The different equipment used for this task are detailed below. Macrographs were taken with a conventional digital camera (Casio model Exilim 12.1 Megapixels, Tokyo, Japan). A Celestron Handheld Digital Pro 5MP microscope (Torrance, CA, USA), equipped with white LED-type light, was also used to take photographs with medium magnification to see the general appearance of the materials. Samples of timber were polished using SiC sandpapers 220#, 500# and 1000#.The moisture content was verified using a resistance xylohygrometric equipment, Seltar model HGD-1 (Barcelona, Spain), which ratifies the normative prescriptions and allows determining the moisture content during the tests [7,24]. Before the test specimens were prepared, the wood had to be kept in a wet chamber with an atmosphere with a relative humidity of 65 ± 5% and at a temperature of 20 ± 2 °C until a constant mass was achieved [25].

To observe the microstructure of the steels an optical microscope was employed. The micrographs were taken with a ZEISS Axiovert 100A microscope (Jena, Germany). The microscope has a digital camera, CCD model OPTIKAM B9 3MP (Ponteranica, Italy), controlled through Optika Vision Lite v. 2,12 (Ponteranica, Italy) software. Previously, the samples were prepared using SiC sandpapers 220#, 500# and 1000# and a final polishing with a 0.1 µm diamond dispersion for metallographic observation. In order to observe the microstructure of the steels, a metallographic attack using 3% nital (nitric acid in ethanol), during 20 s, was carried out. The previous tensile tests on the steels were carried out on a Universal Pressing Machine Model multi-test equipment, Nestor 10 Tn. max. (Madrid, Spain).

### 2.3. Timber Description

**Figure 2:**
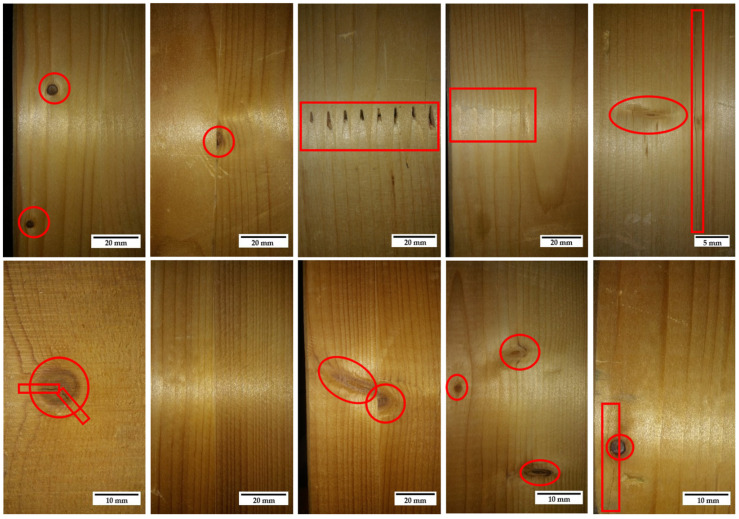
Most common defectology of the wood used in the tests.

**Figure 3:**
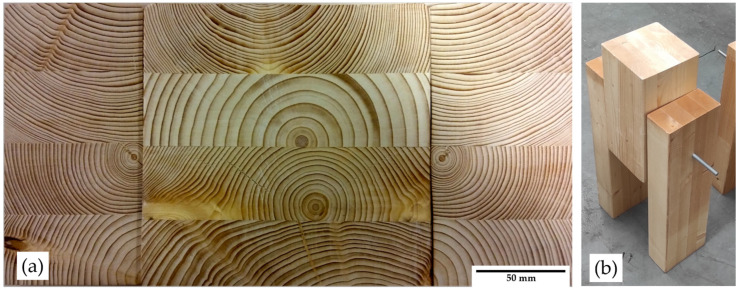
Configuration of the timber (GL24h) used for the tests: (**a**) detail of the polished sample, upper part of the test specimen, and (**b**) specimen for the test.

The wood considered for the research is based on structural use criteria [26]. A glued laminated timber type, normally used in structural applications, was selected [21,22]. This type of timber has advantages over the sawn timber. For example, the beam and column sections are not limited by the diameter of the tree trunk. In addition, the use of homogeneous glued wood (GLh) has become generalized in the structural field due to a series of advantages: it can be produced in almost any shape and size, the effect of imperfections compared to individual pieces of solid wood is reduced and it allows large structural components to be produced economically (without the limitations of lumber due to the size of the log) [27]. Figure 2 shows some examples of defectology admitted by standards and common in this type of wood. As can be seen, the size of this type of defect is small and does not affect the glued assembly. An example of the panel configuration for the test is shown in Figure 3.

**Figure A3:**
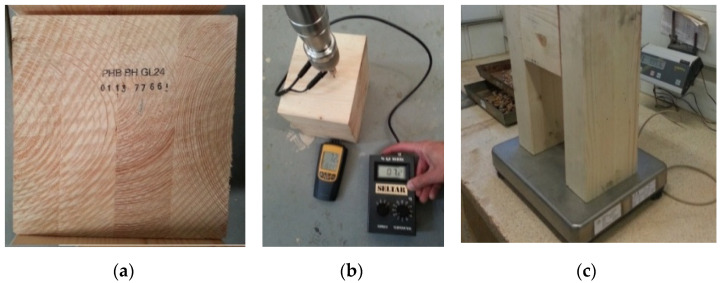
Characterization of the wood: (**a**) trace stamps on the wood from tests; (**b**) humidity control; (**c**) weight control in each specimen and by extension density.

The mechanical properties of the timber used in the tests, certified as resistance class GL24h, are listed in Table 1 according to the standard [25]. Timber was supplied by Noritec Holzindustrie GmbH (Feistritz, Austria). Supplied with resistant class certificates registered by the Austrian laboratory Holz Cert and carrying out verifications of humidity and weight control (with this calculation of the real density) as shown in Figure A3. The thickness of each glued sheet is 40 mm and its width is 80 mm. Figure 3a, with aspect ratios and tolerances in accordance with the standard [25]. The mean value of the density has been calculated from the weight of each timber specimen and its volume was 436.09 kg/mm^3^. The density allows to determine the resistance to crushing in the timber using Equation (5) [4].

(5) $$f_{h,0,k}=0.82\times \left(1-0.01\times d\right)\times \rho_{k}$$

### 2.4. Characterization of the Connections and Fixings

The material used for the connections are structural steel. The nominal diameters (d) are 10 mm. Eurocode 5 does not establish specific references for the type of material to be integrated in these fasteners, so ferritic steel was selected.

**Figure 4:**
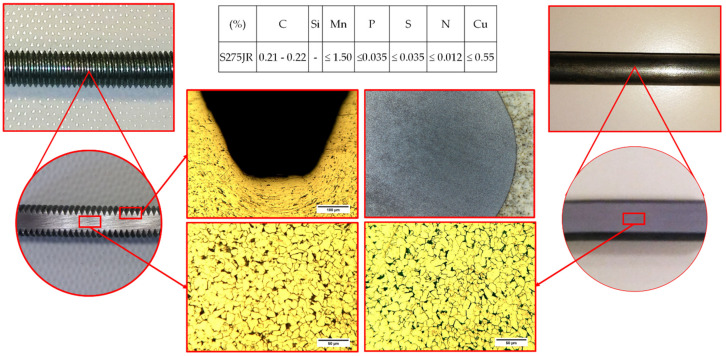
Resume of metallurgical study before tests for both cases: threaded and simple rod.

All the connections were made of ferritic steel graded as S275JR (non-alloy quality structural steel). S275JR grade steel is used in long and flat product formats to manufacture welded and riveted steel structures, as well as in the construction of machinery in low load parts (bushings, shafts and semi-shafts). The steel has been characterized according to the standard by means of simple tensile tests [28]. Its average mechanical properties were obtained from tensile tests with the following values: Young’s modulus Es = 210 GPa, Poisson’s ratio ν = 0.3, yield strength f_y_ = 348 MPa and ultimate strength f_u_ = 496 MPa. Mention that the threaded connections are zinc-plated to prevent corrosion but that it does not affect their mechanical behavior. Figure 4 shows the details of the metallurgical study of steel for both cases: flat and threaded rod.

The washers under the nut must have a side (if they are square) or a minimum diameter equal to 3d and a minimum thickness of 0.3d [7]. Another restriction that is considered is the compressive stress under the washer, which should not exceed 3f_c,90,k_ [7]. The washers dimensions were 2.54 ± 0.01 mm thick with an external diameter of 29.75 ± 0.01 mm and internal diameter of 10.4 ± 0.01 mm.

### 2.5. Experimental Procedure

**Figure 5:**
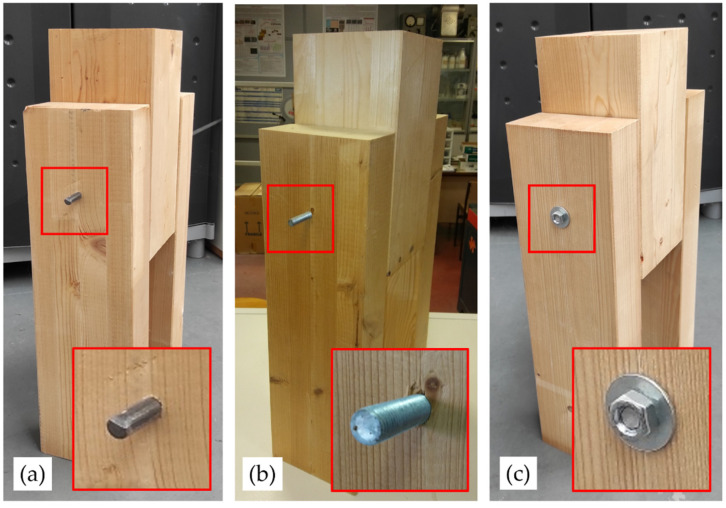
Example of test specimens: (**a**) flat dowel; (**b**) threaded dowel; (**c**) threaded dowel with nut-washer fixing.

The samples were defined from three repetitions of specimens with the similar characteristics. In this work, three types of samples of connections with flat dowel, threaded dowel and threaded dowel with nut-washers were selected in order to compare the effect of fixing with nut-washer (Figure 5).

To identify each specimen, the notation (PXXX) will be used, where “P” is indicative of specimen and “XXX” the number of specimen and its corresponding test, so this is destructive. For the samples, understood as a grouping of same specimens, the process will be similar: with the notation (MXX), where “M” is indicative of sample and “XX” is the sample number.

The M25 corresponds to specimens P134, P135 and P136 with flat dowel, the M29 specimens to P56, P59 and P60 with threaded dowel without fixing at ends, the M03 specimens P01 P14 and P26 with threaded dowel and nut-washer fixing.

### 2.6. Development of Test and Load Program

A Codein 30 t multi-test press, Model MCO-30 (Fuenlabrada, Madrid, Spain) was used. The tests were controlled by constant load speed, with a 300 kN load cell. The standards [29] establish that the test must be programmed based on an estimated maximum load (F_est_) for the type of joint to be tested. The estimated load must be adjusted using the preliminary tests. The work started from the analytical calculation values (F_v, Rk_) determined according to [7] to define the F_est_ values. During the tests, the maximum load (F_max_) can deviate by more than 20% from the estimated value (F_est_). In such a case, the estimated load must be adjusted for successive tests. The standard allows the values already determined to be used, but adjusting the deformations and the crush modulus, which is why in the tests carried out the variables calculated in the continuous load cycle (exceeding the stop of 30 s by 10% of the estimated load) have been determined by calculating on the maximum load (F_max_) of the test.

The charging procedure described in [29] must be followed: Once the estimated load (F_est_) has been set, the load is gradually applied until reaching 40% of this value (0.4·F_est_) and is maintained for 30 s. Afterwards, it is reduced to 10% (0.1·F_est_) and held for another 30 s to increase the load again until reaching the estimated final load or a displacement of 15 mm. The speed of application of loads must be slow, and the standard considers that up to 70% of F_est_ can be applied a constant rate of load or displacement that corresponds to 0.2 F_est_ per minute ±25%. Reached at 0.7F_est_, a constant scrolling speed must be applied; the objective is that the final load (or 15 mm displacement) is achieved in an additional test time of 3 to 5 min.

**Figure 6:**
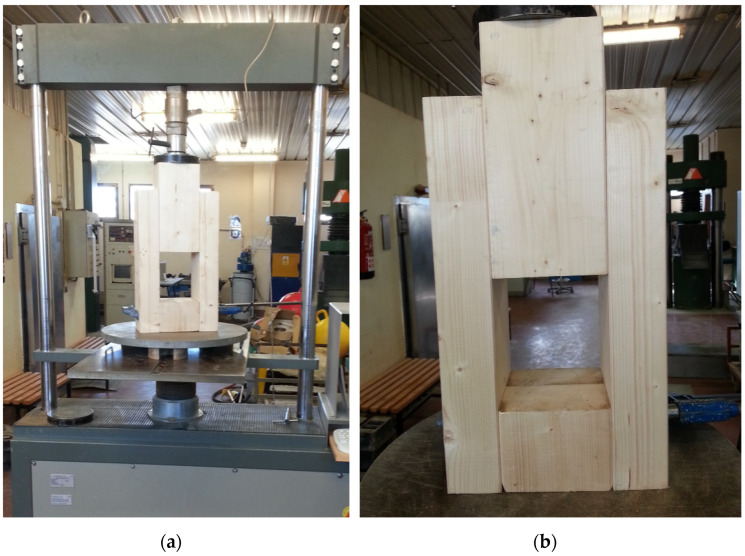
Testing machine with specimen arrangement: (**a**) placement in the press and (**b**) detail of the specimen to be tested.

**Figure 7:**
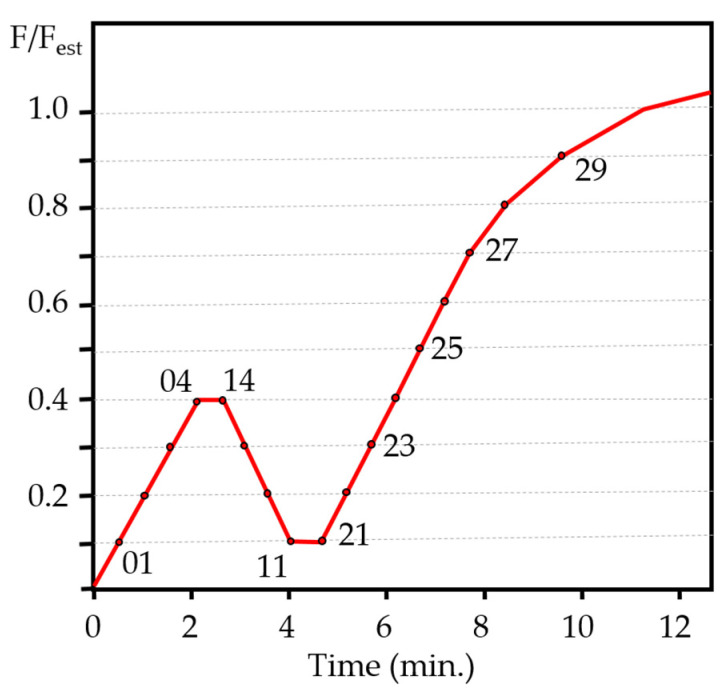
Load cycle applied during the tests.

This assumes that the duration of the test is between 10 and 15 min. Other references [30] indicate test durations of 300 ± 120 s (5 ± 2 min) for the determination of some physical and mechanical properties, although the test program will adhere to the indications of the standard [29], given that the time set in this (15 min) is closer to the static working conditions of the structure. Figure 6 shows an arrangement of the specimen on the testing machine and the load cycle in Figure 7.

## 3. Results

### 3.1. Evolution of the Plastic Moment in the Dowel Hinge with the Load

To calculate the F_est_ load in the test cycle, it has been utilized the equations proposed by [7] where it is necessary to determine the plastic moment M_y,RK_, based on Equation (6).

(6) $$M_{y,\mathrm{Rk}}=0.3\times f_{u,k}\times d^{2.6}$$

**Figure 8:**
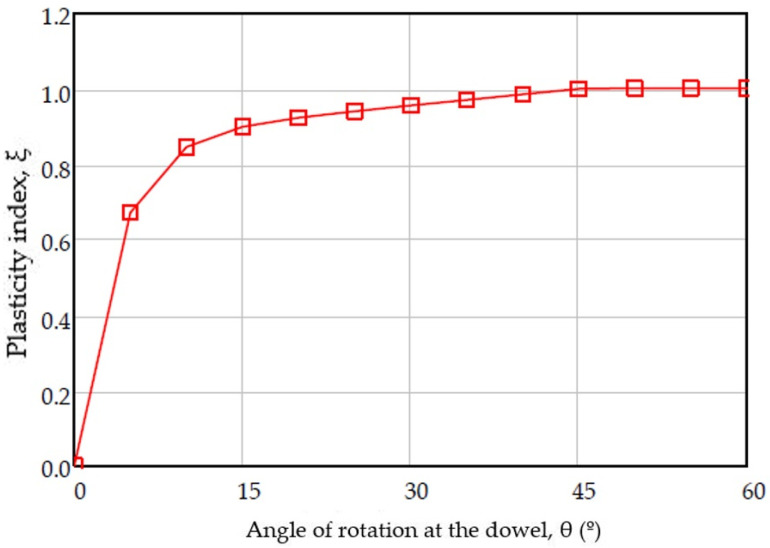
Plasticity index evolution.

There are other studies that establish a relationship between the yielding moment in bending of the dowel and the displacement, relating them to the yield stress (f_y,k_), according to Equation (7). In this equation a coefficient is established as indicates in Equation (8) which is the ξ plasticity index of the flat dowel that was established experimentally by [31] as a function of the angle of the deformed dowel (Figure 8) and therefore of the displacement.

(7) $$M_{y,\mathrm{Rk}}=\frac{\xi \times f_{y,k}\times d^{3}}{6}$$

(8) $$\xi =mín\{\begin{array}{c} \left(0.866+0.00295\times \theta \right)\times \left(1-e^{\left(\frac{-0.248\times \theta }{0.866}\right)}\right)\\ 1 \end{array}$$

**Figure 9:**
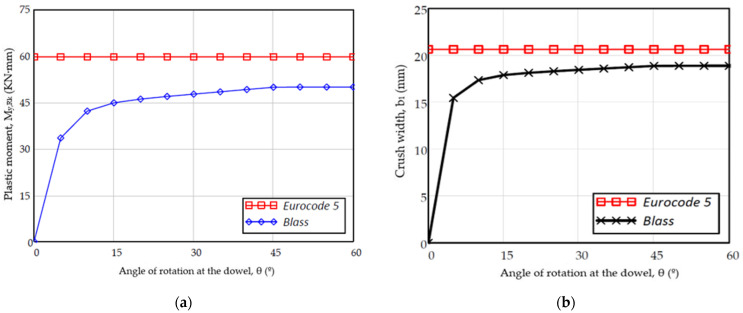
(**a**) Plastic moment versus angle variations in the dowel joint; (**b**) crushing width versus angle variations in the dowel joint.

When the plastic hinge of the dowel is developed with a 45° angle of rotation of the dowel, with large displacements, a limit value of the plasticity index is taken (ξ = 1.0), which places the index on the side of safety. The defined plastic moment equations [7] do not consider the joint displacement, so a load-displacement relationship cannot be established. However, Blass [30] contemplates null moments if there is no displacement and progressively increase to asymptotic values with the plastic bending moment proposed by the Eurocode when the displacements are significant (Figure 9a).

On the other hand, knowing the angle of the rotation of the dowel (θ), the Eurocode proposal identifies constant crush widths as the displacement propagates, while Blass [31] proposes a variation of the crush width (b_1_ + b_2_ according to Figure A1) linked with the angle of the dowel. This would seem the real behavior that the joint should have, because, when the dowel is in its initial position, the crush width cannot be maximum (Figure 9b).

The equations of Blass [31] identify a resistant moment being on the safety side while facilitating the relationship between the crush width, the angle of rotation of the dowel and the displacement (u).

### 3.2. Study of the Crush Widths

**Figure 10:**
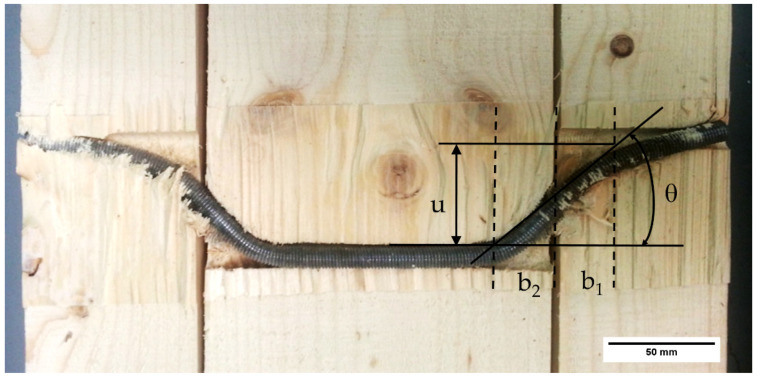
Relationship between displacement and crush widths with the formation of the plastic hinge.

In our experimental results, special attention has been paid to the measurement of the crush width b_1_, since it largely determines the load capacity of the joint. Regarding the crush width, there are two factors to consider. The first that the widths b_1_ and b_2_ increase with the hinge joint angle of the dowel (θ). With small displacements, the angle (θ) and the total width of crushing (b_1_ + b_2_) are smaller [32]. Initially, it propagates from the corners of the wood causing a cutting effect and increases with the displacement (Figure 10).

**Figure 11:**
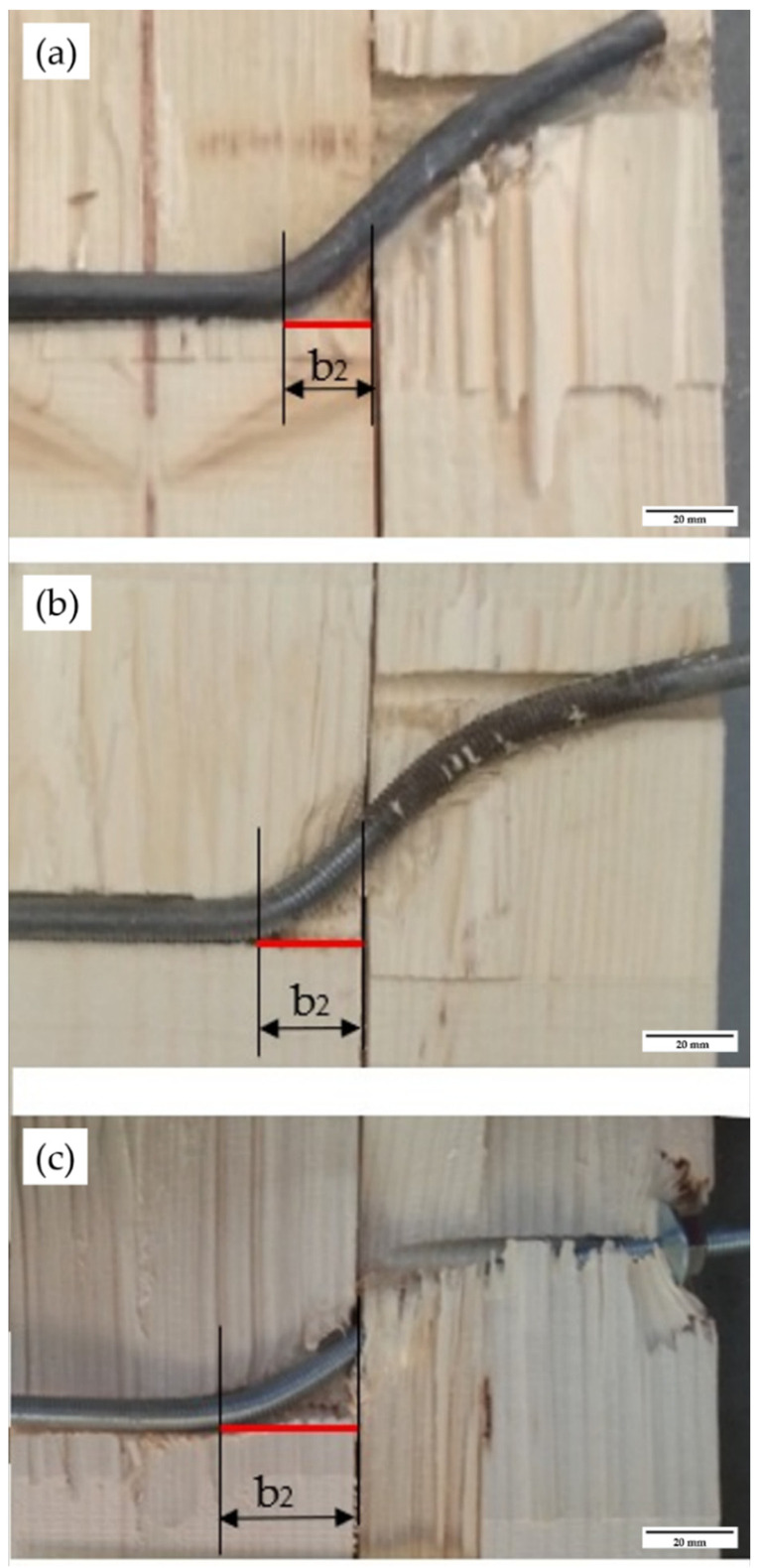
Detail of the area and width of the crushing: (**a**) plain dowel P136; (**b**) threaded dowel P060; (**c**) threaded dowel with end fixings P026.

The second factor is that the crush widths b_1_ and b_2_ are also affected by the fixing of the dowel. In Figure 11, it can be seen that the crush width b_2_ is variable with the type of dowel inserted. Thus, in the flat dowel types (M25) the crush width takes average values of 20 mm, increasing slightly up to 24 mm in the case of the threaded dowel without fixing (M29) and with values greater than 32 mm in the case of threaded dowel with nut-washer fixings at its ends (M03). The width value does not present significant variations in the flat dowels of the same sample with variations of ±2 mm.

If the experimental values are compared for the three cases studied according to Figure 11, this coincides sensitively with the flat dowel pitch. However, it can be seen that the roughness of the threading and even more so the nut-washer fixing at the ends favours the rope effect by introducing greater crush widths. This indicates an increment in the load capacity as the axial force F_ax,Rk_ increases.

### 3.3. Load Study vs. Displacement

**Figure 12:**
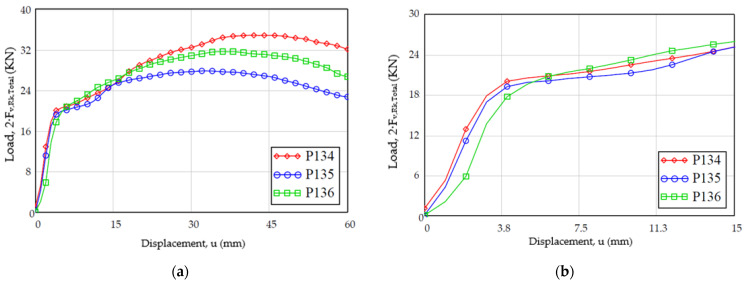
Evolution of the experimental load-displacement for the specimens of the sample [M25]: (**a**) with displacement up to 60 mm; (**b**) detail with displacement up to 15 mm according to standard.

On the other hand, regarding the evolution of the load versus the displacement, the results are shown in Figure 12. The evolution of the displacement follows a trajectory in accordance with the test steps (a first load where the stiffness decreases with the displacement and immediately a plateau area), taking into account that the graph must be corrected eliminating the initial displacement due to the clearance between the dowel and the dowel. In both graphs, it can be observed that the results present a similar aspect, where after the plateau area the load values decrease.

**Figure 13:**
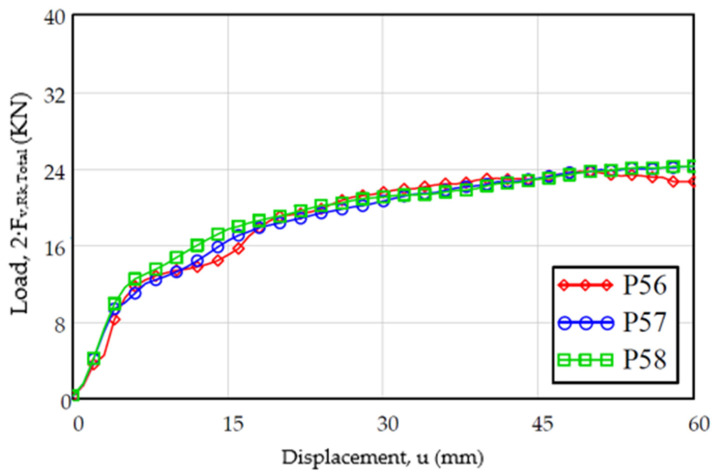
Evolution of the experimental load-displacement capacity of the sample (M29).

In the initial phases, with displacements less than 1 mm, settlements occur with very low loads around 5000 N, identified at an inflection point in the Load-Displacement curve. Figure 13 shows an evolution of the load vs the displacement that presents positive slopes even with displacements greater than 15 mm. This suggests that the thread is embedded within the timber fibers, which favors a rope effect distributed throughout the length of the thread in the entire hole surfaces as indicated in previous studies [32]. However, with small displacements it can be seen that the load capacity in threaded dowels is lower than in the case of flat dowels, most likely due to the difference of the net diameters (d_int_) that in the threaded dowels is reduced by the presence of thread.

**Figure 14:**
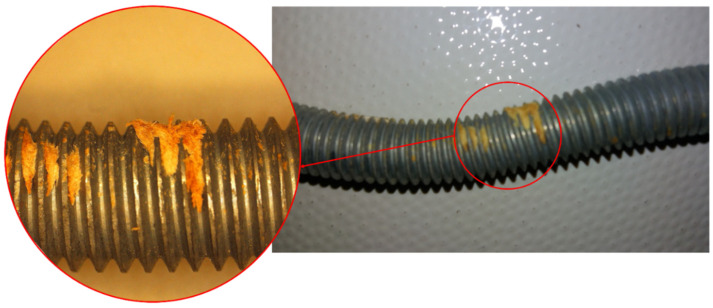
Detail of the embedment that occurs between the thread and the timber.

**Figure 15:**
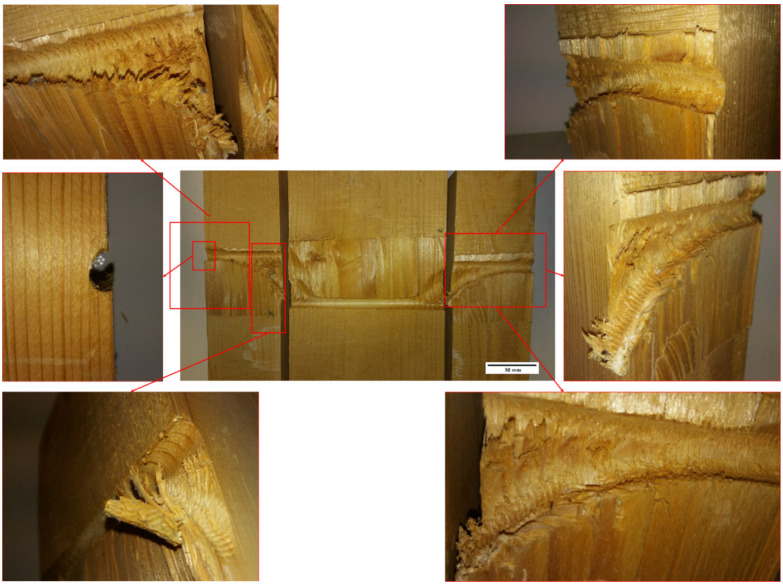
Condition of the timber after the test with dowel without washer nut fixing ends. In the external areas, the wood has crushed due to the dragging of the threaded dowel and when the displacement grows, the threaded dowel is embedded in the timber, causing clawing or gripping and contributing to the rope effect.

Figure 14 shows how the timber fibers are embedded in the thread. This embedding effect prevents the free movement of the dowel thanks to the threading, producing an anchorage. Figure 15 shows a section of the test piece with a threaded dowel, in which various details can be seen, such as crushing by dragging, breaking of the wood and anchorage or grip of the threading, all of them contributing to the rope effect.

**Figure 16:**
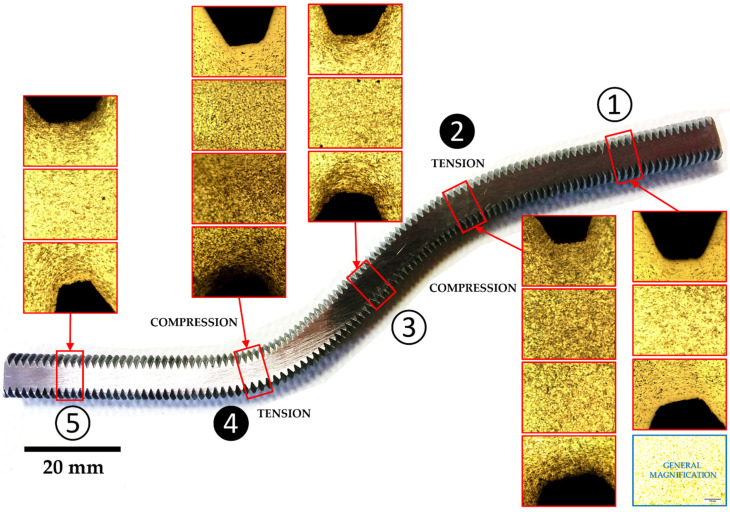
Nital attack over a threaded dowel without nut-washer fixing. The most attacked areas in the steel show more accumulated defects (vacancies, dislocations, etc.) which indicates the areas most stressed under tension (2 and 4) and where the failure will probably occur.

It is well known that stress areas will be much more susceptible to corrosion attack. When corrosion attack begins, it is not limited only to the grain boundaries, but also to the accumulation of defects generated by effect of bending during test. Then, the level of corrosion attack depends on the defects introduced by the deformation. Higher corrosion coincides with higher stress (mainly tensile stress or “tension”, due to proliferation of defects—vacancies, interstitials, dislocations, etc.). Any additional manipulation (testing) of the original product (the dowel) can increase the level of internal structural stress, increasing the defects level, and therefore corrosion in these areas. This phenomenon can be manifested by a uniform chemical attack of the part with nital (3%, 20 s), where the areas subjected to tension present a greater darkening compared to the areas that are in compression or less deformed. This fact can be observed in Figure 16, which shows microstructural details of the threaded dowel. The most deformed areas under tension (2 and 4—black marks) are indicated compared to the rest of the steel dowel. The area marked as 4 shows a greater intensity attack and it would probably be where the failure will occur.

**Figure 17:**
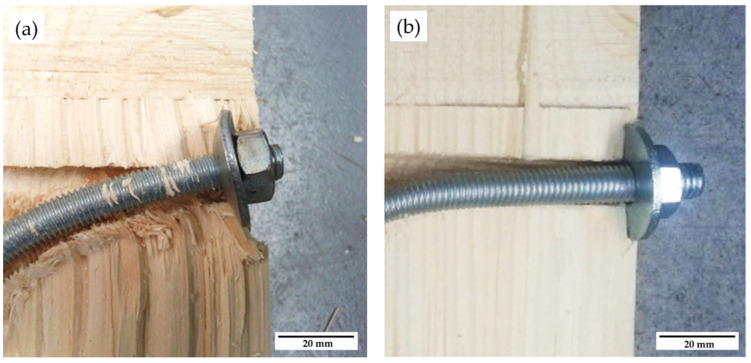
Detail of the final state of the dowel ends fixed with a nut-washer: (**a**) P014 specimen; (**b**) P001 specimen.

In the tests of specimens with nut-washer fixings, the prestressing effect on the contact surface of washer and timber must be considered. Prestressing is important because it allows an initial axial load to be established in the dowel. In addition, it sets a value of the axial force more precisely and consequently allows an initial numerical value of the rope effect component to be set. Additionally, it improves the friction effect in the timber-timber interfaces that has been neglected [4]. The prestressing calculation has been based on [33]. In the Figure 17, it can be seen how the displacement of the joint can end up damaging the washer itself and the timber surfaces in contact with it.

Taking into account the final state in dowel ends fixed with a washer nut, it is important to limit the mode of failure that can occur at this end. It can be advisable to choose a washer that maintains its initial rigidity to achieve a uniform crush on the contact surface with the timber. The washer must be strong enough to avoid excessive deformations, in steel, especially when compared to the timber. Given that the timber is softer than the steel, it is advisable to design the washer in a way that limits the crushing of the timber when the displacement occurs.

**Figure 18:**
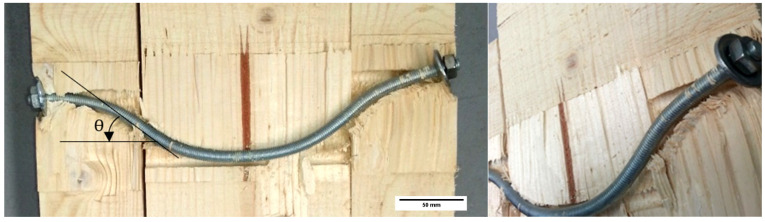
State of deformation in the nut-washer threaded dowels.

With the previous premises, a simplified geometric model can be proposed that relates the variation in length (ΔL) that could be produced by the crushing of the washer against the timber as a function of the internal deformation in the dowel. In the case of the connection with nut-washer fixings (M03), the position of the plastic hinge is not established in such a defined way in specific sections of the dowel (Figure 18), as it was in the case of a flat dowel without fixings (which does not takes into account the axial effect). The deformation of the dowel is exposed to combined bending-tensile stresses derived from the presence of the washer (which increases the axial component), which causes progressive deformation angles and alters the crush zones, being wider with greater displacements (u).

**Figure 19:**
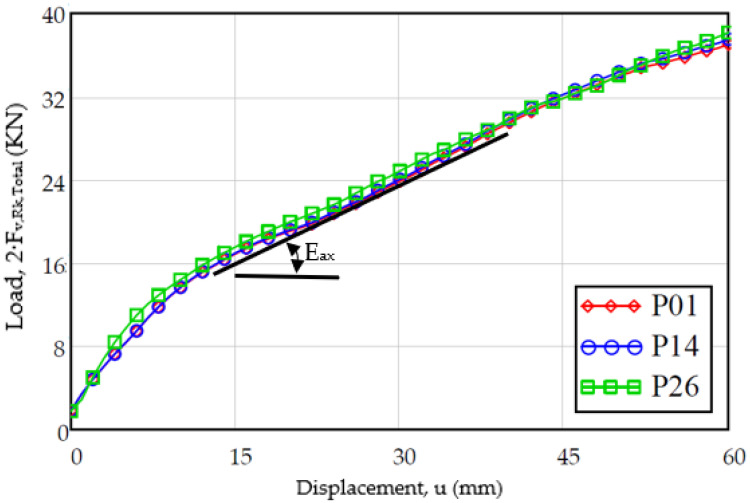
Improved load capacity in connections with nut-washer fixation at their ends (M03).

Figure 19 shows the tests carried out with the sample (M03), where the ends of the dowel were fixed with a nut and washer. Surely, this is one of the reasons why these specimens present a continuous increase with no apparent limit in the load capacity, replacing the plateau area of flat dowels without end fixings with nut-washer.

In these tests, a first phase can be seen with a behavior similar to that of the previous tests, but that changes significantly as the displacement is greater. While in the specimen with a flat surface dowel, a plateau zone was identified, in this case the slope of the curve continues to remain positive, thereby increasing the load capacity as displacement increases. For this reason, it is proposed to determine a slope in the plastic behavior of the hinge (E_ax_), which in this case is 500 N/mm, which has been calculated in the displacement range between 15 and 45 mm.

**Figure 20:**
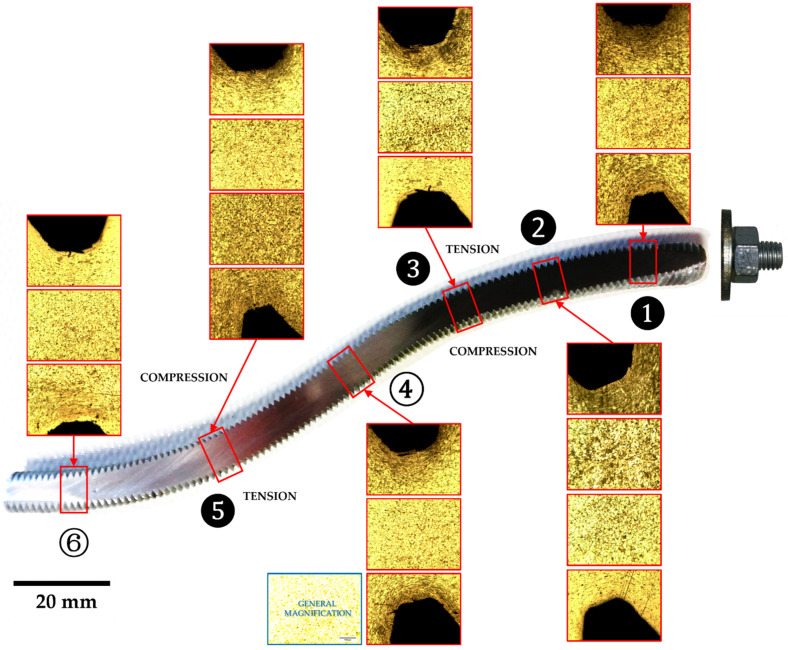
Nital attack over a threaded dowel with nut-washer. Unlike the previous one, the stresses that are produced are lighter (1, 2, 3 and 5) and the also there are less defectology in the area where they are attached to the nut-washer (upper right).

In Figure 20, a nital 3% attack during 20 s was done on threaded dowel with nut-washer. The presence of a nut-washer fixings produces a different distribution of stresses along the dowel when it is tested. The series with this type of elements shows an increase in the corrosion areas (1, 2, 3 and 5, black marks) along the dowel. The most important fact an increase of dark areas where the dowel is attached to the nut-washer. The stress is distributed more uniformely along the dowel thanks to nut-washer. This distribution is quite different to case of threared dowel without nut-washer (Figure 16) and corresponds to the aforementioned. The dowel deformation becomes affected by a combined bending-tensile stresses, due to the presence of the nut-washer, causing a progressive deformation and altering the crush width.

**Figure 21:**
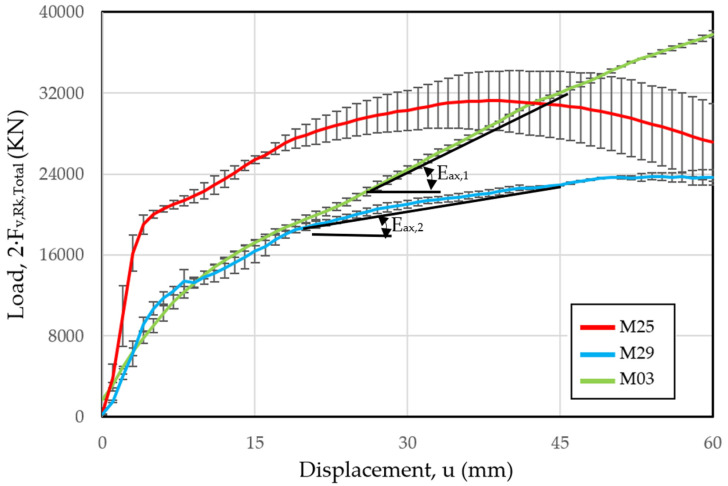
Evolution of the experimental load-displacement capacity for the mean values of the sample with flat dowel (M25), with threaded dowel (M29) and with threaded dowel with end fixation (M03).

Figure 21 shows the average load capacity values in each of the samples, considering the different tests with their tolerance margin between minimum and maximum values. In it, the variations of the three specimens of the sample (M25) can be compared, where it presents a relatively high load capacity with small displacements. These are due to the fact that the diameter of the dowel is identical to the diameter of the hole, which implies a higher initial stiffness and consequently a greater load capacity with small displacements. However, once the plastic hinge appears, its stiffness drops and there is even a loss in load capacity.

Comparing the load capacity in the case of threaded dowels with same net sections as the ones without thread, it can be seen that in the first stages both maintain a similar load capacity. However, as the displacement increases, the dowel with nut-washer fixings diverges from the one without fixings, which shows that the rope effect acquires greater importance the greater the displacement, especially after the plasticization process and the formation of the plastic hinges. Both have positive slopes, which shows that there is a rope effect in both. In the case of the threaded dowel, due to the embedment of the timber fibers, while in the case of the dowel with a nut-washer, the previous effect is superimposed, plus that of the fixings at the ends.

Taking into account the proposed parameter of slope in plastic behavior of the joint (E_ax_), the value of E_ax_ = 500 N/mm can be treated as the superposition of two effects: the threading with a magnitude of E_ax,1_ = 100 N/mm plus that of the nut-washer fixing with a magnitude of E_ax,1_ = 400 N/mm.

On the other hand, in the connection with a flat dowel (M25), it is more difficult to identify a constant dependent line and the range in which it should be calculated is not so characteristic. This indicates that despite being a flat dowel, the rope effect can also be considered like a friction between steel and timber, which varies with the degree of tightening or expansion of the dowel within the timber [34].

Therefore, it seems logical to think that the parameter E_ax_ should be developed in more detail as a function of multiple variables, such as: the diameter of the dowel and the factors that contribute to the rope effect. This provides that the plastic hinges have been formed, not contributing significantly when the dowel has not formed the plastic hinge.

## 4. Discussion

When comparing the crush stress (Equation (5)) from the characteristic density (at its 5th percentile value) and the diameter of the dowel [4,7], they propose to establish the calculation of the load capacity (according to Equation (2)), depending on whether characteristic density values are used (ρ_g,k_ = 385 kg/mm^3^), standard mean (ρ_g,m_ = 420 kg/mm^3^) or the experimental mean of the timber tested (ρ_g,m,tested_ = 436.09 kg/mm^3^). Hereinafter, mean values will be calculated from the experimental density.

Table 2 shows the values of F_v,Rk_ according to analytical equations compared with the test load capacity results. The value of the test load is difficult to assess in practice because the test results in slopes with increasing load capacity. Especially in those specimens more exposed to the rope effect.

It should be noted that the exposed techniques involve comparing an analytical value based on the allowable stresses with a practical value based on displacement limited to 15 mm, regardless of the geometric dimensions of the connection. It can be verified that the load capacities of the test are on average 290.9% higher than those indicated in the standard with characteristic values and a 273.4% with values of average density determined with the timber from the tests. The variation in F_ax,Rk_ and F_ax,R,m_ is practically negligible. This could lead to the assumption that the standard is on the safety side, but it is really counterproductive because it induces an increase in the diameter of the dowel beyond what is necessary and consequently a weakening in the useful section of the timber [35]. Considering the axial load capacity in the analytical calculation, it can be identified what is a very small value that is practically negligible when in the tests it can be seen that it is a variable incremental value with the displacement, especially once the plasticizing hinges have been formed. These results confirm the need to rethink the rope effect as a function of the displacement (u) in the analytical equations of Eurocode 5.

## 5. Conclusions

The analytical equations developed by Johansen in 1949 are the fundamental basis for the study of the dowel type joints resistance. These equations have been assimilated by several national standards and were developed to be used in dowels without fixings at their ends. This work demonstrates that a vector equilibrium diagram is needed to:

- Correctly consider the effect of using nut-washer fixings at their ends which generates the so-called rope effect.
- Limit the possibilities of failure in the timber region under the nut compression.

The influence of the rope effect over the joint resistance is dependent on its contribution as a force vector and on the state of plasticization in the dowel, being necessary to know the relationship between displacement and rigidity of the assembly to assess the contribution of the rope effect F_ax_. The rope effect is variable, increases with the displacement and requires different equations to:

- Consider its influence depending on its origin (the friction between the dowel and timber hole surfaces, the insertion of the dowel thread in the wood or the presence of the nut-washer fixings at their ends).
- Allow the superposition of its effects with the other resistance mechanisms present in the connection.

Increasing the diameter of the dowel is one of the options that achieves the most significant resistance improvements, but it implies to increase the hole in the timber to contain a bigger diameter dowel, which causes the weakening of the resistance section of the timber and which can trigger very dangerous fragile failure mechanisms, which appear without any warning. Therefore, the equations used to calculate the resistance capacity of the joint should include more parameters. For example, nowadays the equations in the standards only utilise the nominal diameter of the dowel (d), but other more specific parameters should be used, just like:

- The initial hole diameter
- The minimum diameter (or net section) of the dowel if a threaded one is used
- The final diameter, once the assembly has been made, taking into account clearances, and/or the timber crushing under the dowel, especially when the diameter of the dowel is bigger than the hole in which it is inserted.

The standards do not consider all the design factors that can contribute to the resistive capacity increment. For example, the rope effect is one of the factors that can contribute the most to increase the resistive capacity in the joint, because the axial forces that appear in the dowel modify such significant variables as the width of the timber crushed. Additionally, this and other possible design factors should be studied in relationship with the displacement that occurs in the joint, including deviations from the use of mean densities and characteristics that affect crushing stress. The tests show variations in the load capacity F_v,Rk_ with the displacement (u). F_v,Rk_ increases progressively in the initial load phases and due to the rope effect, compared to:

- A constant limit value of the load capacity which is indicated in the standards and
- A constant limit value of the displacement (u = 15 mm) in the joint to be used in the tests.

The indications included in the standards and these constant limit values do not match with the results of the experimental tests done in this work. This study introduces parameters that relate F_v,Rk_ with the displacement, integrating more precisely the rope effect as an E_ax_ force. Derived from this, it should be convenient to establish:

- The displacement limits of the joint in relationship with the joint size, because a constant displacement limit (u = 15 mm) may be excessive in small-dimension joints and, on the contrary, very restrictive in large joints
- The rope effect resistive capacity increments, especially in joints using nut-washer fixings.

## Figures and Tables

**Table 1:** Mechanical Properties of the timber used of strength class GL24h (*Picea abies*) [25].

Properties	Symbol	Value
Bending strength (N/mm^2^)	f_m,g,k_	24
Tensile strength parallel to grain (N/mm^2^)	f_t,0,g,k_	19.2
Tensile strength perpendicular to grain (N/mm^2^)	f_t,90,g,k_	0.5
Compressive strength parallel to grain (N/mm^2^)	f_c,0,g,k_	24
Compressive strength perpendicular to grain (N/mm^2^)	f_c,90,g,k_	2.5
Shear strength parallel to grain (N/mm^2^)	f_v,g,k_	3.5
Modulus of Elasticity parallel to grain (N/mm^2^)	E_0,g,m_	11,500
Modulus of Elasticity perpendicular to grain (N/mm^2^)	E_90,g,m_	300
Shear modulus (N/mm^2^)	G_r,g,m_	65
Characteristic density (kg/mm^3^)	ρ_g,k_	385
Mean density (kg/mm^3^)	ρ_g,m_	420

**Table 2:** Comparison of load capacity according to standards and test results with displacement at 15 mm.

	Eurocode 5				Experimental	Safe Factor	
Serie	2·F_v,Rk,EC5_ (N)	2·F_v,R,m,EC5_ (N)	F_ax,Rk_ (N)	Test Sample	2·F_v,R,Exp_ (N) (u = 15mm)	F_v,Rk,EC5_ /F_v,R,Exp_ (%)	F_v,R,m,EC5_ /F_v,R,Exp_ (%)
M25	8714.2	9274.4	0	P134 P135 P136	25,173.5 25,116.7 25,968.2	288.9 288.2 298.0	271.4 270.8 280.0
M29	5767.2	6137.9	0	P056 P059 P060	15,022.7 16,517.4 17,577.0	260.5 286.4 304.8	244.8 269.1 286.4
M03	5779.4	6150.1	6.1	P001 P014 P026	16,966.5 16,963.5 17,524.9	293.6 293.5 303.2	275.9 275.8 285.0

## Nomenclature

Lower case symbols and abbreviations	
a_1 (2)_	width external crushing in the interaction between timber and dowel (mm)
b_1(2)_	internal crush width on timber side (middle) piece (mm)
d	nail diameter (mm).
d_h_	nail head diameter (mm).
d_int_	net diameter (mm)
f_ax,k (m)_	characteristic (mean) value of the tear-off resistance in the nose piece (N/mm^2^)
f_h,1 (2),k (m)_	characteristic (mean) resistance to crushing for the side (middle) piece of timber (N/mm^2^)
f_head,k_	characteristic value of nail head punching resistance (N/mm^2^)
f_m,g,k_	bending strength of timber (N/mm^2^)
f_c,0 (90),g,k_	compressive strength parallel (perpendicular) to grain of timber (N/mm^2^)
f_t,0 (90),g,k_	tensile strength parallel (perpendicular) to grain of timber (N/mm^2^)
f_v,g,k_	shear strength parallel to grain of the timber (N/mm^2^)
f_y,k_	yield strength of steel (N/mm^2^)
f_u,k_	ultimate strength of steel (N/mm^2^)
u	displacement (mm)
t_pen_	penetration length in the nose piece or the length of the corrugated portion in the nose piece (mm)
t_h_	thickness at the head of the piece (mm)
t_1 (2)_	thickness of timber side (middle) piece (mm)
t_h_	thickness at the head of the piece (mm)
Uppercase symbols and abbreviations	
E_0 (90),g,m_	modulus of elasticity mean parallel (perpendicular) to fibers of timber (N/mm^2^)
E_ax_	slope in the plastic behavior of the hinge (N/mm)
E_s_	modulus of elasticity of steel (GPa)
G_r,g,m_	shear mean modulus of timber (N/mm^2^)
F_est_	estimated load (KN)
M_y,Rk_	plastic moment (KN·mm)
F_ax,Rk (m)_	characteristic (mean) load capacity upon removal of the fixing element (KN)
Greek symbols	
ρ_g,k, (m)_	characteristic (mean) density of timber (kg/mm^3^)
ξ	plasticity index of the dowel
ν	poisson’s ratio of steel
β	crush strength ratio (f_h,2,k_/f_h,1,k_)
θ	hinge joint angle (°)
ΔL	length of embedment of washer in wood (mm)

## Appendix A

Figure A2 shows examples of actual structures in the historic set of the docks of the Canal of Castile, the Chapel of Santa Ana (Valladolid), the Real Monastery of Santa Clara (Tordesillas) and the Historical Garden of the Béjar Forest, all in Spain, using dowel type joints with washer-nut endings. These structures have been studied and documented by several authors [36,37].

Figure A3 shows the characterization of the properties of the timber, the control in the specimen assembly process and the moisture and density control by weight in each specimen.
